# Pretest probability assessment derived from attribute matching

**DOI:** 10.1186/1472-6947-5-26

**Published:** 2005-08-11

**Authors:** Jeffrey A Kline, Charles L Johnson, Charles V Pollack, Deborah B Diercks, Judd E Hollander, Craig D Newgard, J Lee Garvey

**Affiliations:** 1 Department of Emergency Medicine, Carolinas Medical Center, Charlotte, NC, USA; 2 Computational Biology Program, BreathQuant Medical Systems Inc, Charlotte, NC, USA; 3 Department of Emergency Medicine, Pennsylvania Hospital, Philadelphia, PA, USA; 4 Department of Emergency Medicine, University of California at Davis, Sacramento, CA, USA; 5 Department of Emergency Medicine, Hospital of the University of Pennsylvania, Philadelphia, PA, USA; 6 Department of Emergency Medicine, Oregon Health & Science University Medical Center, Portland, OR, USA

## Abstract

**Background:**

Pretest probability (PTP) assessment plays a central role in diagnosis. This report compares a novel attribute-matching method to generate a PTP for acute coronary syndrome (ACS). We compare the new method with a validated logistic regression equation (LRE).

**Methods:**

Eight clinical variables (attributes) were chosen by classification and regression tree analysis of a prospectively collected reference database of 14,796 emergency department (ED) patients evaluated for possible ACS. For attribute matching, a computer program identifies patients within the database who have the exact profile defined by clinician input of the eight attributes. The novel method was compared with the LRE for ability to produce PTP estimation <2% in a validation set of 8,120 patients evaluated for possible ACS and did not have ST segment elevation on ECG. 1,061 patients were excluded prior to validation analysis because of ST-segment elevation (713), missing data (77) or being lost to follow-up (271).

**Results:**

In the validation set, attribute matching produced 267 unique PTP estimates [median PTP value 6%, 1^st^–3^rd ^quartile 1–10%] compared with the LRE, which produced 96 unique PTP estimates [median 24%, 1^st^–3^rd ^quartile 10–30%]. The areas under the receiver operating characteristic curves were 0.74 (95% CI 0.65 to 0.82) for the attribute matching curve and 0.68 (95% CI 0.62 to 0.77) for LRE.

The attribute matching system categorized 1,670 (24%, 95% CI = 23–25%) patients as having a PTP < 2.0%; 28 developed ACS (1.7% 95% CI = 1.1–2.4%). The LRE categorized 244 (4%, 95% CI = 3–4%) with PTP < 2.0%; four developed ACS (1.6%, 95% CI = 0.4–4.1%).

**Conclusion:**

Attribute matching estimated a very low PTP for ACS in a significantly larger proportion of ED patients compared with a validated LRE.

## Background

Despite its importance, pretest probability assessment remains a relatively imprecise and inferential process, sometimes referred to as the "doctor's best guess" [1,2]. Previous authors have broadly defined pretest probability as the clinician's estimate of the probability of disease from the patient's words, physical findings, risk factors, and exposures, rendered prior to knowledge of objective test results [3,4]. Presently, the most widely recognized quantitative method of determining pretest probability employs the logistic regression equation [5-10]. The logistic regression equation can be used to estimate the probability of a target disorder in individual patients by assembling and weighing the importance of characteristics found to be predictive of that disorder in an equation. The predictive power of the characteristic is reflected in the magnitude of its coefficient in the equation. The equation can then be solved explicitly to provide a point estimate of probability based upon the sum of the coefficients and the intercept. From the perspective of probability estimation for acute disease, one of the main drawbacks to logistic regression equations is that they seldom output a pretest probability in the very low (0–5%) range. In particular, this very-low pretest probability range is extremely important in the context of evaluating a patient with chest pain in the emergency department, because this is the range where the clinician must decide whether or not to use the resources required for formal testing by a chest pain protocol [11].

We hypothesize that accurate pretest probability assessments can be obtained by matching an individual patient to a group of previously studied patients who shared the same clinical characteristic, and determining the percentage of these previously studied patients who had the outcome of interest. This hypothesis proposes a method of inference that differs substantially from the logistic regression equation. Instead of treating each clinical characteristic as an independent value and adding up the coefficients, we propose a system that forces the probability to be computed from a dependent set of clinical characteristics. In other words, all chosen characteristics of a patient of interest must be matched before a previously studied patient is eligible for pretest probability computation.

In this report, we derive and test a computerized method to estimate the pretest probability of acute disease by matching the clinical characteristics (or *attributes*) of a patient of interest to an identical profile of attributes shared by a group of patients with known outcomes contained in a large derivation database. The derivation database contains prospectively collected clinical attributes of emergency department patients who were evaluated for acute coronary syndrome and for whom the results of diagnostic testing plus 30-day follow-up are known. First, we used the classification and regression technique to select the variables for attribute matching in the derivation database. We then wrote a computer program to perform the attribute matching procedure. Next, we tested the computerized system in an independent validation population of emergency department patients. The focused question of this work was how often and how accurately the novel system would produce a pretest probability that is below the test threshold. The test threshold represents the point estimate of pretest probability derived from a formula that considers the risks of false positive testing versus the risk of untreated disease [12]. The formula predicts that a patient with a pretest probability below the test threshold treatment will not benefit from further testing.

## Methods

### Model derivation

The reference database used for model derivation and as the source for pretest probability assessment was drawn from the multicenter internet tracking of acute coronary syndrome (i*tr*ACS*) collaborative conducted in 1999–2001 at 7 hospitals in the United States and one in Indonesia [13]. Local Institutional Review Board approval was obtained to collect these data. An emergency department patient was eligible for enrollment into the registry (Access^® ^Microsoft Corporation) when an emergency physician had sufficient suspicion of acute coronary syndrome to order a 12-lead electrocardiogram. Subsequent decision to perform objective testing for acute coronary syndrome was performed at the discretion of a board-certified emergency physician. While the patients were in the emergency department, a study associate (physician or registered nurse) recorded 70 clinical variables, including the standard historical, risk, physical examination, electrocardiographic and laboratory data using a structured clinical report form. We restricted the reference database to patients aged >15 years, with complete data and 30-day follow-up. With these restrictions, the reference database consisted of 14,796 patients with a mean age of 54 ± 16 years, with 35% identified as Caucasian, 51% female, and an overall 15.8% prevalence of acute coronary syndrome.

For the attribute-matching process, it was necessary to truncate the 70 recorded variables to a smaller subset with statistically significant predictive value. This truncation process was accomplished using classification and regression tree analysis (CART^®^, Salford Systems, San Diego, CA), a form of binary recursive partitioning. Classification and regression tree analysis is a nonparametric method of statistical analysis used to classify observations based on a large number of possible predictive variables, and is well-suited for identifying complex interactions among variables [14]. This methodology has been described previously [15]. A quantitative ranking of the variables (i.e. relative importance) was generated by CART^®^, based upon the frequency and importance of a given variable in the tree building process. Classification and regression tree analysis identified eight variables as having discriminatory value for the prediction of acute coronary syndrome: 1. Age (divided into four groups, <35, 35–38, 39–50, and >50 years), 2. Gender, 3. Race (white or asian, and nonwhite, non-asian), 4. Patient report or physician observation of sweating with symptoms, 5. Patient report of a prior history of coronary artery disease or myocardial infarction, 6. Chest pain worsened with manual palpation on physical examination, and 7. Electrocardiographic manifestation of ST segment depression > -0.5 mm (-50 μV) in any two leads, 8. T wave inversion > -0.5 mm in any two electrocardiographic leads. These eight variables were then denoted as "attributes" in the matching process. In the matching sequence, age produced a four-way split and the other 7 attributes were binary splitter nodes, allowing a maximum of 4*2^7 ^or 512 unique matching permutations (termed *attribute profiles*).

### User interface

**Figure 1:**
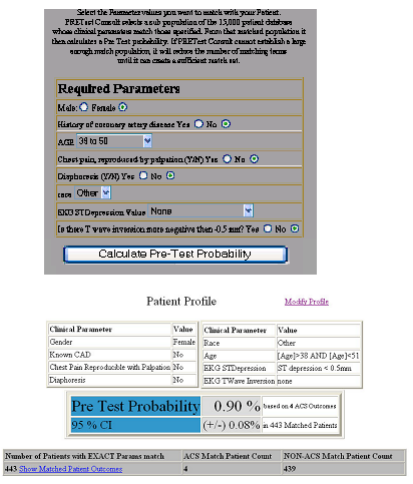
Screenshot of the user interface designed to perform the attribute matching process. The application is preloaded with the attributes of a hypothetical patient with chest pain, and shows the calculated pretest probability.

**Figure 2:**
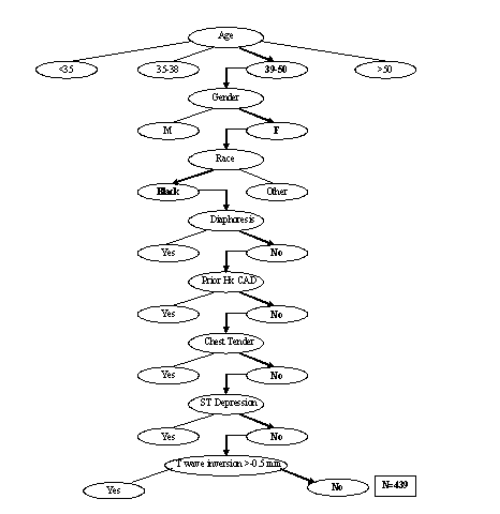
Illustration of the attribute matching algorithm for the hypothetical patient whose profile were input in Figure 1. The patient is a 41 year old African-American woman who presents without diaphoresis, has no history of coronary artery disease and chest pain that is not reproducible with palpation (Chest Tender), and has no ST depression or T wave inversion >0.5 mm (2 leads) on electrocardiography. This match process returned 439 patients from the derivation database. Four of 439 had acute coronary syndrome within 45 days, yielding a pretest probability of 0.9% for this hypothetical patient.

To facilitate the process of attribute matching, an author (CLJ) wrote computer source code in visual basic with standard query language subroutines to allow the user to mouse click the eight matching attributes into fields displayed on the screen of a personal computer or handheld personal digital assistant (Figure 1). After the eight attributes were populated and submitted for a new patient, the computer program then extracted from the reference database only those patients with the attribute profile exactly matching that of the patient of interest (Figure 1). Thus, the attribute-matching pretest probability estimate for any new patient was based upon assigning the patient to one of 512 exact, unique attribute profiles. Each profile returned a specific number of patients from the database, and no one patient could be assigned to more than one profile. Figure 2 illustrates the logic the attribute matching process for a hypothetical patient.

### Validation testing

The validation population was a new set of emergency department patients who underwent evaluation for suspected acute coronary syndrome including 30 day outcomes based upon telephone contact and medical chart review. These patients were enrolled at two hospitals, the University of California, Davis and the Hospital of the University of Pennsylvania in Philadelphia from 2001–2002. In addition to other clinical data, all patients included in the validation population had the variables required for the attribute matching process as well as the logistic regression. The choice of diagnostic testing to rule out acute coronary syndrome was at the discretion of a board-certified emergency physician who had unrestricted (24-hour, 7 day) access to the resources of a tertiary academic hospital, including serial biochemical markers, exercise treadmill testing, nuclear imaging, and cardiac catheterization. At both centers, these variables were prospectively collected and recorded using an identical data collection form, which was completed in the emergency department, prior to knowledge of diagnostic testing outcome. Patients with an initial 12-lead electrocardiogram that demonstrated 1 mm (+100 μV) ST segment elevation in two or more leads were analyzed separately. Patients were excluded if data required for either method of pretest probability computation were absent, or if the patient were lost to follow-up.

### Logistic regression equation

To provide a benchmark decision rule for comparison of diagnostic utility, we computed the pretest probability estimate from the logistic regression equation described by Selker and colleagues (the acute cardiac ischemia-time insensitive prediction instrument, ACI-TIPI). The variables and coefficients of this method have been published previously. This equation can output a maximum of 128 unique probability estimates [16-18].

### Outcome definition

In both the source database and in the validation testing, the definition of acute coronary syndrome included any of the following events, occurring within 30 days of initial evaluation: 1. Acute myocardial infarction, defined by the European Society of Cardiology/American College of Cardiology consensus recommendations [19], 2. Performance of percutaneous coronary balloon angioplasty, with or without intracoronary stent placement, 3. Coronary artery bypass grafting, 4. Death within 30 days after initial evaluation.

### Computation of test threshold

The focused question was how often and how accurately each method could produce a low enough pretest probability to potentially forestall a formal protocol to evaluate for acute coronary syndrome. This was done by defining the testing threshold as described by Pauker and Kassirer [see additional file 1] [12]. This estimate was 2.0%. This value is consistent with data in published literature indicating a 0.5% to 3.1% probability of acute coronary syndrome 30 days after negative evaluation in a chest pain evaluation protocol [11,20-25]. For the purpose of evaluating diagnostic accuracy of the attribute matching method, 2.0% is analogous to the positive cutoff in a diagnostic test. Patients with pretest probabilities below 2.0% are hereafter referred to as having "very low risk" of developing acute coronary syndrome. The boundaries defining low-, moderate-, and high-risk categories were based upon pretest probability intervals that are consistent with published practice patterns for admission status of emergency department patients [11,21,24].

### Statistical analyses

Confidence intervals for proportions were computed from the exact binomial distribution using Wilson's method (StatsDirect, v 2.2.8) [26]. The median pretest probability estimates between the two methods were compared using the Mann-Whitney U test; Spearman's rank correlation coefficient statistic (rho) was used to test for concordance. The overall diagnostic performance of both methods of pretest probability assessment were determined by rounding the point estimate of pretest probability to the nearest whole percentage and construction of a receiver operating characteristic curve, where all patients with higher pretest probabilities were considered test positive. The overall diagnostic performance was assessed as area under the receiver operating characteristic curve computed by the trapezoidal rule with 95% confidence intervals computed using the Wilcoxon estimate. Gaussian curve fitting was performed in Sigmaplot v. 7.0, SPSS company, Chicago, IL.

## Results

The validation population was drawn from 8,120 ED patients who were prospectively evaluated for possible acute coronary syndrome. One thousand sixty-one patients (13%) were not included in the primary analysis for the following reasons: 713 (8.9%) patients had 1 mm or more ST elevation in two or more leads on electrocardiogram; 77 (0.9%) did not have all required data fields for the logistic regression equation; 271 (3.3%) had no ACS-defining event and were lost to 30-day follow-up. The remaining 7,059 patients in the validation population had a mean age of 54 ± 15 years; 57% were female, 38% were Caucasian; 68% percent had a chief complaint of chest pain, but 8% had no chest pain. All patients had two blood cardiac markers drawn separated by 8 hours. Troponin I concentrations measured using the AxSYM^174; ^system (Abbott Laboratories, Abbott Park, IL). 2780 (39%) patients underwent additional cardiac-specific diagnostic testing, including treadmill electrocardiography in 745 (10%), treadmill echocardiography in 105 (2%), or a nuclear cardiac imaging study in 1135 (16%) and cardiac catheterization in 795 (11%). All 7059 patients had 30 day follow-up performed. Six hundred-one of 7,059 patients, or 8.5%, were diagnosed with acute coronary syndrome within 30 days, including 348 acute myocardial infarctions (5%) and 92 deaths. The absence of acute coronary syndrome was established in the remaining 6,458 patients based upon scripted 30-day telephone follow-up with the patient.

**Figure 3:**
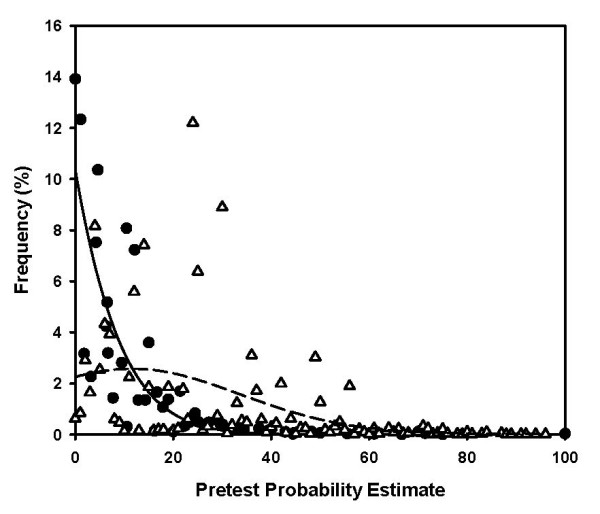
Plot of the frequency of each discrete pretest probability as a function of the actual pretest probability, estimated on the validation population using two methods. A Gaussian curve was fitted to the attribute matching points (solid line) and the logistic regression points (dashed line). The median pretest probability estimate from attribute matching was 5.5% (1^st^–3^rd ^quartiles: 1.0–10.5%), versus 24.0% (1^st^–3^rd ^quartile 10.0–30.0%) for the logistic regression method (P < 0.001, Mann Whitney U). Because of rounding, each point could represent more than one unique match profile.

The two methods generated contrasting sets of pretest probabilities in the validation population. The attribute matching method utilized 267 of the 512 possible unique pretest probability estimates to yield a median pretest probability value of 5.5% (1^st^–3^rd ^quartiles: 1.0–10.5%). The median match size (denominator) used to compute the pretest probability from the attribute matching method was 192 patients (1^st^–3^rd ^quartiles: 94 to 340), and 6,104 of the 7,059 (87%) pretest probability estimates had a match size ≥ 50 patients. The top 10 most frequent matching profiles are shown in Table 1. Four of the top ten profiles generated pretest probabilities less than 2.0%. In contrast, the logistic regression equation produced 96 unique pretest probability estimates, yielding a significantly higher median pretest probability value of 24.0% (1^st^–3^rd ^quartile 10.0–30.0%) compared with the median from attribute matching (P < 0.001, Mann Whitney U). Figure 3 plots the frequency of each whole percentage pretest probability as a function of the rounded pretest probability estimate value for the validation population and plots an overlying best-fit Gaussian curve for both methods (Y = a*exp [-0.5((X-Xo)/b)^2^]. With the attribute matching method, the frequency of the pretest probability result tended to be inversely related to the magnitude of the pretest probability estimate with two-thirds of its pretest probability estimates less than 10%. In comparison, logistic regression categorized 54% of the pretest probability estimates between 10 and 30%.

**Table 1:** Top 10 most frequently matched attribute profiles in the validation population

Patient Count*	**%PTP**	**Age**	**Sex**	**Race**	**Sweating**	**Coronary Artery Disease**	**Reproducible Chest Pain**	**ST Depression**	**T-Wave Inversion**
467	5	>50	F	Non-white, non-Asian	None	No history of CAD	None	None	None
354	1	39–50	F	Non-white, non-Asian	None	No history of CAD	None	None	None
353	6	>50	F	White, Asian or other	None	No history of CAD	None	None	None
272	12	>50	M	White, Asian or other	None	No history of CAD	None	None	None
237	6	>50	F	Non-white, non-Asian	None	No history of CAD	None	None	> -0.5 mm
225	1	39–50	M	Non-white, non-Asian	None	No history of CAD	None	None	None
217	10	>50	M	Non-white, non-Asian	None	No history of CAD	None	None	None
194	4	39–50	M	White, Asian or other	None	No history of CAD	None	None	None
160	1	39–50	F	White, Asian or other	None	No history of CAD	None	None	None
157	0	<35	F	Non-white, non-Asian	None	No history of CAD	None	None	None

Abbreviations: %PTP-Percentage pretest probability rounded to nearest whole number, CAD-Coronary artery disease, M-male, F-female

*Refers to the number of patients in the validation set (out of 7,059) matched to the profile

**Figure 4:**
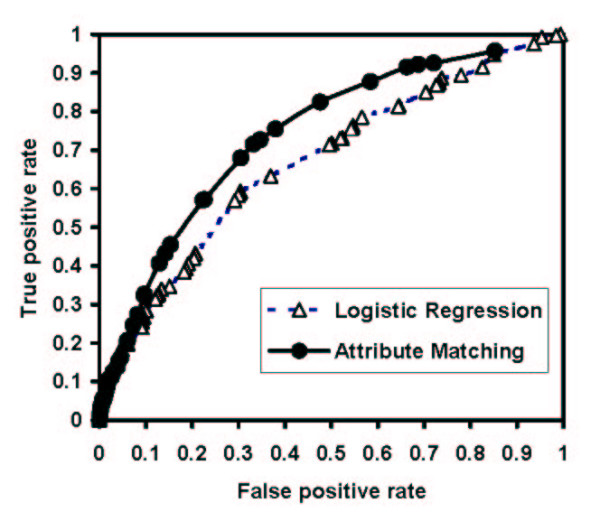
Receiver operating characteristic curves for two methods of pretest probability assessment. The area under the curve measurements were 0.74 (95% CI 0.65 to 0.82) for the attribute matching curve and 0.68 (95% CI 0.62 to 0.77) for the logistic regression method.

To determine the overall diagnostic performance of the two methods, the pretest probability estimates were rounded to the nearest whole percentage, and receiver operating characteristic curves were constructed using each whole percentage as cutoff points on the curves (Figure 4). At each whole percentage, patients with higher pretest probability values were considered "test positive." Both methods demonstrated only fair overall discriminatory value; the area under the curve measurements were 0.74 (95% CI 0.65 to 0.82) for the attribute matching curve and 0.68 (95% CI 0.62 to 0.77) for the logistic regression method.

We then sought to aggregate the pretest probability estimates into four clinically relevant categories, (Table 2): 1. Very low-risk (<2.0% pretest probability of acute coronary syndrome, the testing threshold subgroup); 2. Low-risk category (2.0 -< 10.0%), 3. Moderate (10%–30%), and 4. High risk (>30%). To generate these categories using the attribute matching method, a stable pretest probability was required to have a match size ≥ 50 patients (maximum very low-risk 95% CI = 0–10%). Table two shows pretest probabilities computed in the validation population using both methods. The two methods demonstrated modest concordance (Spearman's rho = 0.69). The attribute matching method generated a match size >50 in 87% of subjects. Attribute matching yielded a very low pretest probability with an adequate match size in 1,670 or 23.7% of all patients, and among these patients, 28/1,670 or 1.7% (95% CI = 1.1 to2.4%) developed acute coronary syndrome, three of whom died during follow-up (3/1,670= 0.2%, 95% CI = 0 to 0.5%) and one of whom developed an acute myocardial infarction. The logistic regression method produced a very low pretest probability in 244 or 3.6% of all patients, and among these patients, 4/244 or 1.6% (95% CI = 0.4 to 4.1%) developed acute coronary syndrome, one of whom died (1/244 = 0.4%, 95% CI = 0 to 2.2%) and none had myocardial infarction. The sensitivity and specificity of the very low-risk designation for the detection of acute coronary syndrome at 30 days was 95.3% and 25.4% for attribute matching, versus 99.3% and 3.7% for the logistic regression method.

**Table 2:** Comparison of pretest probability estimates for acute coronary syndrome using two techniques in a validation population.

	**Category of Pretest Probability**				
	Very Low (<2%)	Low (2 to <10%)	Moderate (10 to 30%)	High (>30%)	Indeterminate*

**Attribute Matching**					
Number with ACS†	28	189	250	9	125
Total in category‡	1670	2953	1453	28	955
Prevalence of ACS	1.7%	6.4%	17.2%	32.1%	13.1%
% of all patients assessed	23.7%	41.8%	20.6%	0.4%	13.5%
**Logistic Regression**					
Number with ACS†	4	59	272	266	NA
Total in category‡	244	1356	3813	1646	NA
Prevalence of ACS	1.6%	4.4%	7.1%	16.2%	NA
% of all patients assessed	3.5%	19.2%	54.0%	23.3%	NA

*Estimates from attribute matching with match size <50. Does not apply to logistic regression.

†Total number of patients with ACS = 601

‡Total number patients in validation study = 7059

Abbreviations: ACS acute coronary syndrome.

**Figure 5:**
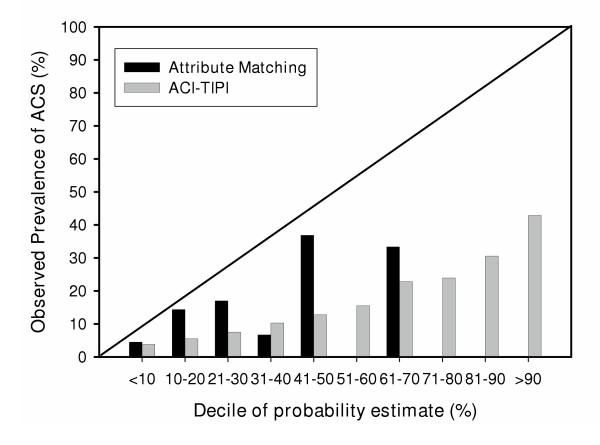
Plot of the actual prevalance of acute coronary syndrome as a function of the predicted probability of acute coronary syndrome in 7,059 patients without ST segment elevation. Data are aggregated within deciles. The diagonal line represents the ideal relationship between predicted probability versus observed probability.

Figure 5 plots the actual, observed prevalence of acute coronary syndrome as a function of the decile of the predicted probability by each method in the 7,059 patients who did not have ST segment elevation. Neither method closely followed the diagonal line that represents the performance of the hypothetically perfect pretest probability system. However, the graph suggests that compared with attribute matching, ACI-TIPI produced a more reliably linear relationship between the observed versus predicted prevalence of ACS.

Although the purpose of this report was to focus on the low-risk chest pain patients (i.e., the 7,059), we believe it is useful to compare the performance of the attribute matching system and the ACI-TIPI equation in the population that included the 713 patients who had pathological ST segment elevation on the initial 12 lead electrocardiogram. In this higher risk subgroup, 202/713 (28%) patients were ultimately diagnosed with an acute coronary syndrome. The attribute matching system categorized 78 of these 713 patients as having a pretest probability <2.0%, and among these patients, 4 (5.1%) were diagnosed with acute coronary syndrome. The ACI-TIPI system categorized 33 of these 713 patients as having a pretest probability <2.0% and none of these patients had an outcome of acute coronary syndrome. Thus, if ST segment elevation patients were included in the validation set (N = 7,772), attribute matching would have produced a sensitivity of 96% and specificity of 24.6%. The ACI-TIPI equation would have produced a sensitivity of 99.5% and a specificity of 3.9%. The false negative rates would have been 1.8% (95% CI: 1.0 to 3.0%), and 1.5% (0 to 4.0%). These results suggest that attribute matching tends to underestimate the probability of acute coronary syndrome in patients with ST segment elevation on electrocardiography.

The first troponin I concentration, measured in the emergency department, was elevated (>0.3 ng/mL) in 12 of 28 patients who developed acute coronary syndrome after being deemed very low-risk by attribute matching; the initial troponin I concentration was elevated in one of four patients who developed acute coronary syndrome after being deemed very low-risk by logistic regression. The prevalence of acute coronary syndrome in patients with a normal first troponin I concentration plus a very low-risk designation by attribute matching was 16/1,605 or 1.0% (95% CI = 0.5–1.6%).

Both methods produced a stepwise increase in pretest probability with each category. The attribute matching method categorized significantly fewer patients as high-risk (0.4%) compared with the logistic regression technique (23.3%), and the prevalence of acute coronary syndrome was significantly higher in the high-risk group predicted by attribute matching compared with the high-risk group predicted by logistic regression. The sensitivity of the high-risk designation by the logistic regression method was significantly higher (266/601 = 44%) compared with the attribute-matching method (9/601 = 1.5%; 95% CI for difference of 42.5% = 39 to 47%). However, the logistic regression equation categorized 1,380/6,458 or 21% of patients without acute coronary syndrome as high risk compared with 19/6,458 or 0.3% with the attribute-matching method. As a result, the specificity of the high-risk designation by the attribute matching method was significantly higher (99%, 95% CI = 99–100%) than the specificity of the high-risk designation by the logistic regression method (79%, 95% CI = 78–80%).

## Discussion

This report introduces a novel method to estimate of the pretest probability of acute disease based upon computer-assisted, database-derived, attribute matching. The system operates by allowing the clinician to input a predefined set of clinical attributes for a subject for whom the pretest probability is desired. When executed, a computer program queries a large patient database, and returns only the patients who share the identical attribute profile as the new patient being evaluated. The proportion of these attribute-matched subjects who had a clinical outcome of interest comprises the point estimate of the pretest probability. We submit that this process conforms to the prose definition of pretest probability from a clinician's perspective: "the probability of the disease of concern in one patient, based upon prior experience with many patients who had similar clinical characteristics as the patient under consideration" [3,4].

In this report, we used an eight-node matching system capable of generating 512 unique pretest probabilities from a 14,796-patient database. We compared this system to an established logistic regression equation to estimate the probability of acute coronary syndrome. Both methods were tested in a validation study of 7,059 emergency department patients without ST segment elevation who were evaluated for possible acute coronary syndrome, and for whom the 30-day outcome was known. The attribute matching system produced significantly more pretest probability assessments that had a significantly lower median value, and categorized patients as very low-risk for acute coronary syndrome six times more often than the logistic regression equation. The rates of acute coronary syndrome-defining outcomes at 30-days in both very low probability groups were virtually identical at 1.7% (95% CI = 1.1–2.4%) with attribute matching, versus 1.6% (95% CI = 0.4–4.1%) with logistic regression. Among patients with an attribute matching-designated very low pretest probability and one normal initial troponin I concentration measured while the patient were in the emergency department, the rate of acute coronary syndrome at 30 days was 1.0% (95% CI = 0.5–1.6%).

A perfect pretest probability instrument would produce exactly two point estimates of pretest probability: 0% and 100%. In our validation population, this imaginary perfect instrument would yield 6,458 pretest probability estimates equal to zero and 601 estimates equal to 100% in the validation population. This would yield exactly two points on Figure 3, one point at 0% on the X-axis and 91.5% on the Y-axis and another at 100% on the X-axis and 8.5% on the Y-axis. Thus, assuming that its estimates were correct, a real instrument that produces a large cluster of very low pretest probabilities and a small cluster of very high pretest probabilities would begin to model the perfect results. Figure 3 shows that the Gaussian curve fitted to the attribute matching probabilities has an inverse function appearance, with the majority of the estimates occurring in the <5% range, whereas the Gaussian curve fitted to the logistic regression probabilities has a quasi-normal appearance, with the peak of the curve occurring at a pretest probability of 15%.

Physicians are well aware that they can be held negligent for failure to test patients for a clinical picture that provokes the slightest thought of acute coronary syndrome. In the present study, 91% of patients in the validation study had no acute coronary syndrome-defining event within 30 days of initial evaluation (including the "soft" endpoint of revascularization). In a 1997 multicenter study, Graff and colleagues found that only 2.5% of patients evaluated by a chest pain protocol were diagnosed with acute myocardial infarction [27]. In our experience, this "rule-in rate" is declining. We submit that the primary motivation for evaluating many low-risk patients in a chest pain evaluation unit are the clinicians' perceptions of reduced medicolegal risk and the patients' perceptions of increased safety. But approximately 25–30% of these patients will have a false positive or indeterminate provocative test – a result that usually mandates hospital admission, and can lead to performance of coronary angiography negative for coronary artery disease [11,20,22,24,27]. A potential application of the present system would be use of the combination of a pretest probability <2.0%, and one negative biomarker of cardiac ischemia or necrosis, in conjunction with the patient's risk tolerance to prevent unnecessary chest pain protocol evaluation [28]. Reports in the lay and medical literature underscore the desire of patients to become informed and active participants in medical decision-making [29].

The present study does not purport clinical utility of pretest probability derived from attribute matching at this stage. We tested attribute matching against a logistic regression equation that contains different variables, and was not designed to rule out ACS in very low risk patients. We did not test the output of attribute matching against a logistic regression equation that used the exact same variables so this report does not allow a head-to-head comparison of the two methodologies. The present work only tested the accuracy of the derived system by a secondary analysis of prospectively obtained data, albeit a large, two-center sample from opposite sides of the US. The next question to answer is whether the findings are valid in other populations, and if the computerized system adds any value to the implicit estimate of probability from clinicians with variable experience. Attribute matching should also be tested against other published research methods, including other computerized models, Bayesian and neural network systems [6,30]. The heuristic aspects of the attribute matching system warrant more research to define them quantitatively. We recognize that as the number and complexity of the input attributes increases, this will create a more specific and potentially more accurate clinical profile, but at a cost of reduced match size if the reference database remains the same size. In theory, the ideal attribute matching system would allow a very detailed clinical profile to be matched against a tremendously large reference database. In the present work, we used an eight-node attribute profile and a 14,796 patient database that produced a match size of 50 or more in about 87% of patients tested in the validation phase. This match size requirement appears to have resulted in relatively reliable pretest probability assessments in the validation population based upon the categorization data in Table 2. The optimal stoichiometry between the number of matching nodes that can be used to create an accurate pretest probability versus the database size remains uncertain. It could be hypothesized that a larger database and more complex attribute profile would produce a more accurate pretest probability assessment. Finally, the model only had good utility in patients with very low risk for acute coronary syndrome and cannot be used in patients with ST segment elevation. We believe that attribute-matching should be similarly tested for other clinical conundrums, including the evaluation of pulmonary embolism.

## List of abbreviations

ACS: Acute Coronary Syndrome

CI: Confidence Interval

ED: Emergency Department

i*trACS: Internet Tracking of Acute Coronary Syndrome

LRE: Logistic Regression Equation

PTP: Pretest Probability

## Competing interests

JAK and CLJ are both partially employed by BreathQuant Medical Systems, which now markets the ACS software described in this paper.

CDN serves as consultant for BreathQuant on a study that is separate from and unrelated to this paper's focus.

JAK (VP-Medical Director), CLJ (VP-Product Development), and CVP (Scientific Advisor) are all members of BreathQuant's board.

## Authors' contributions

JAK conceived and designed the study, acquired data, assisted in the analysis and interpretation of this data and drafted the paper.

CLJ contributed to study design and made critical revisions to the paper.

CVP acquired data, assisted with the analysis and interpretation of this data and made critical revisions to the paper.

DBD acquired data, assisted with the analysis and interpretation of this data and made critical revisions to the paper.

JEH acquired data, assisted with the analysis and interpretation of this data and made critical revisions to the paper.

CDN acquired data, assisted with the analysis and interpretation of this data and made critical revisions to the paper.

JLG assisted with the analysis and interpretation of this data and made critical revisions to the paper.

## Pre-publication history

The pre-publication history for this paper can be accessed here:



## Supplementary Material

Additional File 1 Test threshold for ACS. Explanation of the formula used to compute the test threshold to rule out acute coronary syndrome. Click here for file
