# Supplementary material for: Pretest probability assessment derived from attribute matching
Source: BMC Med Inform Decis Mak. 2005 Aug 11;5:26. doi: 10.1186/1472-6947-5-26 (PMC1201143; doi:10.1186/1472-6947-5-26)
Supplement: Additional File 1 — Test threshold for ACS. Explanation of the formula used to compute the test threshold to rule out acute coronary syndrome. [file 1472-6947-5-26-S1.doc]

APPENDIX. Computation of test threshold to rule out acute coronary syndrome.

The test threshold defines the pretest probability point below which the patient is at low enough probability for disease that the risks of testing outweigh the benefits of testing. This can be calculated according to the formula described by Pauker and Kassirer.

T_t_ = (P_pos/nd_* R_rx_ + R_t_)/(P_pos/nd_* R_rx_ + P_pos/d_* B_rx_)

Estimates with references for each term for acute coronary syndrome:

P_pos/nd_ = **false positive rate** Based upon information from the Society for Chest Pain Centers, we will assume that the chest pain unit uses exercise treadmill electrocardiography in 70% of patients and treadmill echocardiography in 30% of cases. Thus, the composite false positive rate exercise treadmill testing = 30%.(1) (2-5)

R_rx_ = **risk of treatment**, which is the cumulative risk of a bad outcome from catheterization, followed by treatment with either medical therapy or revascularization.

About 1% of patients undergoing angiography will have a bad outcome, including renal failure requiring dialysis, anaphylaxis, stroke, coronary dissection, or death. Our data show that one-half of patients who undergo angiography with then be treated with a drug or intracoronary stenting (risk of CABG is excluded). These treatments will induce serious complications in a small percentage (≈2%) of patients (e.g., life-threatening drug reaction, coronary artery dissection during angioplasty, or myocardial infarction during stent deploy). Thus, R_rx_ = 1% + 0.5*2% = 2%. However, many patients will not proceed to catheterization, because they will have a nuclear study first. We will assume that the nuclear study has no risk, and that only 30% of patients go on to have a catheterization after the nuclear study. Thus, the 2% estimate must be reduced to 0.6%.

R_t_ = **Risk of the provocative test**. We will assume that 70% of tests are treadmill electrocardiography or echocardiography and 30% are dobutamine-echocardiography. A medline search from 1966 to present using free text search for “treadmill electrocardiography” or “exercise electrocardiography” and “complications,” demonstrated no reports of complications from treadmill testing from emergency department CPUs. However, the occurance of myocardial infarction or ventricular tachycardia was found to be above 1% in two non-ED studies,(6) Two papers report a 1/350 rate of serious complications from dobutamine testing.(7-9). We estimate that 1/2000 patients will arrest have an MI or experience a ventricular arrhythmia where 70% undergo treadmill exercise testing and 30% have dobutamine infused. Thus R_t_ = 0.0005

P_pos/d_ = Composite **sensitivity of the provocative testing**, which we will estimate at 70%(1-5)

B_rx_ = **Benefit of treatment**, the absolute percentage reduction in bad outcomes afforded by treatment in patients with disease. A bad outcome is considered to be a myocardial infarction or death. From a medicolegal and ethical standpoint, the period of responsibility after an ED evaluation for chest pain is assumed to be 90 days. If a bad outcome occurs after 90 days, this is assumed to not inculpate the emergency physician. The highest rate of myocardial infarction or death in the absence of any treatment over one year was assumed to be 30%, as estimated from the placebo arms randomized controlled trails of patients with unstable angina (assumed tantamount to cardiac ischemia).(10;11) A recent multicenter ED trial of sestimibi scanning found a 17% rate of myocardial infarction or death within 30 days among patients with cardiac ischemia who received contemporaneous treatment by a cardiology service capable of interventional treatment.(12) Thus, the best-case B_rx_ (the absolute reduction in progression to death or myocardial infarction over one year) = 30-17 = 13%

T_t_ = (0.3*0.006 + 0.0005)/(0.3*0.006 + 0.70*0.13) = 2.4%, or about 2%

Reference List for Appendix

(1) Diercks DB, Gibler WB, Liu T, Sayre MR, Storrow AB. Identification of patients at risk by graded exercise testing in an emergency department chest pain center. American Journal of Cardiology 2000; 86(3):289-292.

(2) Manini AF, Gisondi MA, van d, V, Schreiber DH. Adverse cardiac events in emergency department patients with chest pain six months after a negative inpatient evaluation for acute coronary syndrome. Academic Emergency Medicine 2002; 9(9):896-902.

(3) Storrow AB, Gibler WB. Chest pain centers: diagnosis of acute coronary syndromes. [Review] [103 refs]. Ann Em Med 2000; 35(5):449-461.

(4) Nichol G, Walls R, Goldman L, Pearson S, Hartley LH, Antman E et al. A critical pathway for management of patients with acute chest pain who are at low risk for myocardial ischemia: recommendations and potential impact.[comment]. Annals of Internal Medicine 1997; 127(11):996-1005.

(5) Graff LG, Dallara J, Ross MA, Joseph AJ, Itzcovitz J, Andelman RP et al. Impact on the care of the emergency department chest pain patient from the chest pain evaluation registry (CHEPER) study. American Journal of Cardiology 1997; 80(5):563-568.

(6) Singh H, Aneja GK, Mehrotra TN, Dwivedi KK, Mitra A, Sood K. Complications of treadmill testing. Journal of the Association of Physicians of India 1996; 44(5):313-315.

(7) Hirano Y, Yamamoto T, Uehara H, Nakamura H, Wufuer M, Yamada S et al. Complications of stress echocardiography. [Japanese]. Journal of Cardiology - Supplement 2001; 38(2):73-80.

(8) Lattanzi F, Picano E, Adamo E, Varga A. Dobutamine stress echocardiography: safety in diagnosing coronary artery disease. [Review] [86 refs]. Drug Safety 2000; 22(4):251-262.

(9) Secknus MA, Marwick TH. Evolution of dobutamine echocardiography protocols and indications: safety and side effects in 3,011 studies over 5 years. Journal of the American College of Cardiology 1997; 29(6):1234-1240.

(10) Theroux P, Ouimet H, McCans J, Latour JG, Joly P, Levy G et al. Aspirin, heparin, or both to treat acute unstable angina.[comment]. New England Journal of Medicine 1988; 319(17):1105-1111.

(11) Lewis HDJ, Davis JW, Archibald DG, Steinke WE, Smitherman TC, Doherty JE et al. Protective effects of aspirin against acute myocardial infarction and death in men with unstable angina. Results of a Veterans Administration Cooperative Study. New England Journal of Medicine 1983; 309(7):396-403.

(12) Udelson JE, Beshansky JR, Ballin DS, Feldman JA, Griffith JL, Heller GV et al. Myocardial perfusion imaging for evaluation and triage of patients with suspected acute cardiac ischemia: a randomized controlled trial.[comment]. JAMA 2002; 288(21):2693-2700.
